# Durability of Mortars with Fly Ash Subject to Freezing and Thawing Cycles and Sulfate Attack

**DOI:** 10.3390/ma15010220

**Published:** 2021-12-28

**Authors:** Monika Jaworska-Wędzińska, Iga Jasińska

**Affiliations:** Faculty of Mechanical Engineering, Kazimierz Pulaski University of Technology and Humanities in Radom, Malczewskiego 29 Street, 26-600 Radom, Poland; i.jasinska@uthrad.pl

**Keywords:** freezing and thawing cycles, frost resistance, low-calcium fly ash, high-calcium fly ash, sulfate attack

## Abstract

Destruction of cement composites occurs due to the alternate or simultaneous effects of aggressive media, resulting in the destruction of concrete under the influence of chemical and physical factors. This article presents the results of changes in the measurement of linear strains of samples and changes in the microstructure of cement after 30 freezing and thawing cycles and immersed in 5% sodium sulfate solution. The compressive strengths ratios were carried out at the moment when the samples were moved to the sulfate solution after 30 cycles and at the end of the study when the samples showed visual signs of damage caused by the effect of 5% Na_2_SO_4_. The composition of the mixtures was selected based on the Gibbs triangle covering the area up to 40% replacement of Portland cement with low and high-calcium fly ashes or their mixture. Air-entrained and non-air entrained mortars were made of OPC, in which 20%, 26.6%, and 40% of Portland cement were replaced with low and/or high-calcium fly ash. Initial, freezing and thawing cycles accelerated the destruction of non- air-entrained cement mortars immersed in 5% sodium sulfate solution. The sulfate resistance, after the preceding frost damage, decreased along with the increase in the amount of replaced fly ash in the binder. Air-entrained mortars in which 20% of cement was replaced with high-calcium fly ash showed the best resistance to the action of sodium sulfate after 30 freezing and thawing cycles.

## 1. Introduction

It is well known that the resistance of cement composites to chemical attack depends on physical factors such as capillary porosity and pore size distribution, which determine the permeability, water absorption and tightness. The main products of sulfate corrosion are ettringite and gypsum. The formation and accumulation of gypsum and ettringite were caused by chemical reactions. Due to immersing, sulfate solution changes the pore structures gradually, which in turn affects the ion transport and then the degradation of concrete structures [1]. One of the ways to protect concrete against sulfate attack is the use of fly ash [2]. The most resistant activity should contain a large amount of C-S-H phase and as little calcium hydroxide and calcium monosulfate and calcium aluminate as possible. Fly ash in the cement binder reduces the amount of calcium hydroxide, as it reacts with active ash compounds in the pozzolanic or pozzolano–hydraulic reaction, which increases the proportion of the C-S-H phase in the cement matrix. The positive effect on the sulfate resistance of composites with the addition of low-calcium fly ash was confirmed in research by Metha [3], who proved that the corrosion resistance of cement composites with the addition of fly ash was influenced by reaction products with sulfate solution. According to some authors [4], the composition of the glassy phase in low-calcium fly ash was partially similar to the composition of the glassy phase in granular blast furnace slag. Therefore, a positive effect of these ashes can be expected on the physical properties and resistance to sulfate aggression on composites containing the addition of these ashes in the binder. The mineral and phase composition of the ashes affected the sulfate resistance of cement composites, but also the amount of Portland clinker replaced with fly ash. Numerous studies were carried out to determine the optimum amount of fly ash in the binder to obtain the best possible resistance to aggressive solutions. It is generally known that replacing 25–30% of Portland with fly ash significantly increases the sulfate resistance. The authors [5] used 20 and 40% fly ash for mortars. The addition of fly ash improved the sulfate resistance; however, the author of [6] suggests that the impact of type-F FA containing high contents of aluminum phase on the sulfate attack on cement mortars at low temperature needs to be individually evaluated. The authors of [6,7] replaced 15, 25–75% of cement with fly ash, respectively; however, they did not present an unambiguous conclusion that the amount of introduced ash alone had a significant impact on the corrosion resistance of cement composites. The authors [8] suggest that replacing 10% of cement with fly ash improves the sulfate resistance of mortars. According to other authors [8] replacing 15–20% of cement with fly ash was not sufficient to increase the durability of concrete after sulfate attack. To ensure satisfactory sulfate resistance, the authors [9] suggest replacing 25–35% of cement with lime ash. Mehta [9] considers that replacing 50% or more of the cement with fly ash, as in HVFAC concrete, can ensure the high strength and durability of concrete.

Air entrainment improves the frost resistance of cement composites, but it is not fully understood whether the air pores formed will have an impact on their sulfate resistance. The additional air voids affect the permeability of cement composites. The air entrainment was changed in the microstructure of the grout, and the insulated air pores were connected by very small capillary pores. According to the authors of [10], air pores are characterized by much lower water absorption than capillary pores, as they generate lower pressure compared to capillary pores. Although the air voids exhibit high permeability, the water absorption of air-entrained cement composites did not increase, provided that they were properly processed in water. Therefore, air entrainment of cement composites may affect their sulfate resistance. In their research, Wong et al. [10] raised the issue of mass transport in air-entrained concrete. In [10], they suggested that the altered microstructure of the slurry around the air pores affects the course of mass transport. According to Santhanam et al. [11,12] aeration of cement mortars did not affect their sulfate resistance. However, the authors of [11,12] based their conclusions on mathematical calculations, and they did not provide any results of expansion studies or changes in the microstructure. The authors of [10] confirmed the necessity to perform tests of air-entrained cement composites to determine their resistance to chemical solutions that have not been implemented so far.

The durability of cement composites was defined as the degree of resistance to freezing and thawing cycles, corrosion, penetration, carbonation, stress and the effects of chemicals, such as sulfate corrosion. Most often, the properties of cement-based composites were tested during the operation of one of the mechanisms of deterioration of durability. However, in existing structures, cement-based composites are often exposed to structural damage due to multiple destructive processes (mechanical, physical, chemical) acting simultaneously or synergistically, which can lead to accelerated failure. Conclusions obtained from laboratory tests of concrete exposed to one type of deterioration do not always correlate with the observations of real structures. Such tests did not take into account the various parameters existing during operation that may affect the mechanisms and kinetics of a given corrosion. Despite the current extensive knowledge of the sulfate corrosion of concrete, cases of premature failure and costly repair of concrete structures exposed to a sulfate environment have been reported [13]. This happens because classical tests of sulfate corrosion do not include such factors as, for example, fluctuations in the pH level, cyclical drying and wetting, which accompany concrete corrosion during operation [13]. The combined frost damage and sulfate corrosion has been reported in several scientific publications. From a practical point of view, such studies can have a significant impact on predicting the durability of cement composites. In the article [14], this problem was highlighted in relation to concrete reinforced with steel fibers. The authors of [14] stated that the freezing and thawing cycles may accelerate the penetration of the solution deep into the sample, but they also raise the issue of lower diffusivity of sulfate ions at reduced temperatures. The author of [15] suggests that the air bubbles in the air-entrained silica fume concrete lower and delay the damage resulting from the crystallization of sulfate salt. The publication [16] indicated the negative impact of combined failure of the durability of concrete with the addition of fly ash. The authors [16] presented the results of tests of concrete that were subjected to freezing and thawing cycles as well as the impact of sulfate and magnesium solutions. The authors of [17,18,19,20] also performed studies of combined frost destruction and sulfate aggression. The authors of [17], replacing 20% of cement with fly ash, obtained better resistance towards the action of combined destruction compared to mortars made of Portland cement. As suggested in [18], replacing 10% OPC with fly ash improved the resistance to combined frost damage and sulfate corrosion. Moreover, as Neville [13] emphasizes, it is important to test the durability of concrete in conditions as close to the natural environment as possible. Therefore, the authors in the article made an attempt to assess the durability of mortars subjected to freezing and thawing cycles, followed by sulfate aggression.

## 2. Research Scope

**Figure 1:**
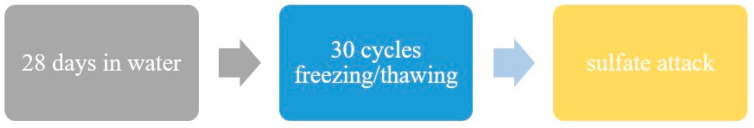
Research plan.

This study aims to determine the effect of fly ashes and their mixture on sulfate resistance and frost action. The damage due to the combined action of these two factors caused cement mortars to be exposed to freezing and thawing, and sulfate attack of 5% Na_2_SO_4_ solution (STANLAB, Lublin, Poland). In the scarce literature, attention was paid to clarifying the sulfate attack process after initial short-term frost action and to the frost action process of mortars with low- and high-calcium fly ash. In this study, the sulfate attack took place after 30 freezing–thawing cycles. The results of the durability of mortars subjected to complex deterioration were compared with the results of mortars with the addition of fly ash (Dyckerhoff Poland, Nowiny, Poland and Górażdże, Rogowiec, Poland) subjected to sulfate corrosion. Thanks to this, it has been possible to determine whether the initial freezing and thawing cycles have an impact on the subsequent sulfate resistance of cement composites. The plan of the research is shown in Figure 1.

## 3. Materials and Methods

### 3.1. Portland Cement

The plan of the experiment included making the Portland cement mortars using the CEM I 42.5R—Portland Cement (CEMI). The chemical composition of Portland clinker (Dyckerhoff Poland, Nowiny, Poland) is presented in Table 1 and the phase composition in Table 2. The results of the phase and chemical composition were provided by the cement producers.

### 3.2. Low- and High-Calcium Fly Ash

**Figure 2:**
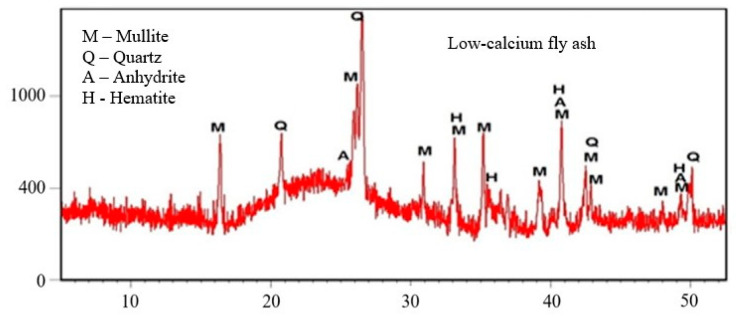
XRD low-calcium fly ash.

**Figure 3:**
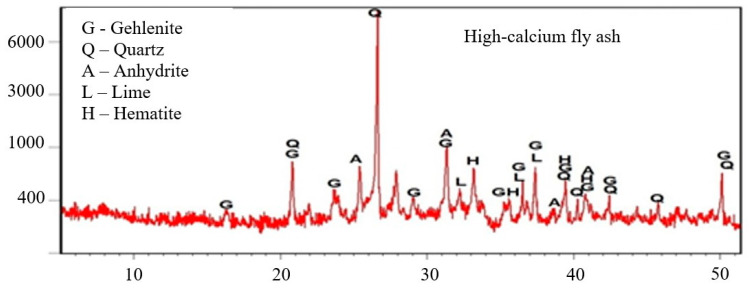
XRD high-calcium fly ash.

The mortars were prepared with the addition of low-calcium fly ash (V) with a loss on ignition of 2.5%, a specific surface area equal to 3233.00 [cm^2^/g] on the Blaine scale and high-calcium fly ash (W) with a loss on ignition of 2.7%. The specific surface area of the high-calcium fly ash was equal to 2860.00 [cm^2^/g] on the Blaine scale. The chemical compositions of the fly ash are presented in Table 3. The results of the XRD fly ash phase composition test are presented in the diffraction diagram (Figure 2 and Figure 3).

### 3.3. Composition of Mortars

**Figure 4:**
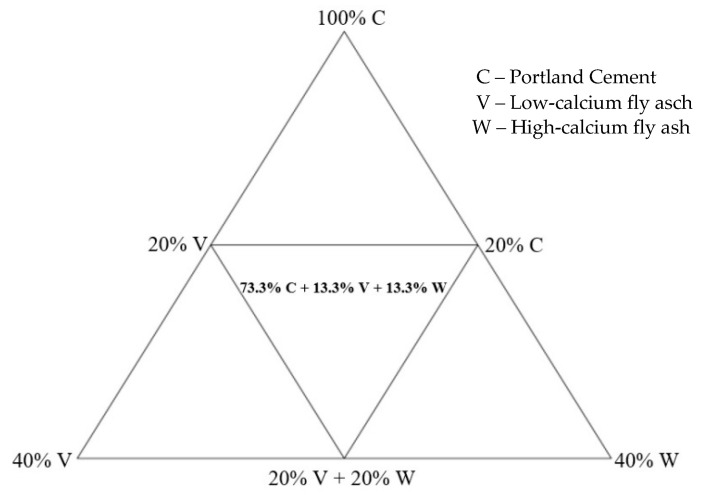
Plan of cement mortar binder mixtures.

The design of the composition of mortars was based on the Gibbs triangle. The composition of the mortars used for the testing was developed on the basis of a simplex experiment plan, which was used in the case of testing the properties of the mixture depending on its composition. In order to be able to apply the experimental design for ternary mixtures, the area where a maximum of 40% of Portland cement was replaced with one of the fly ashes or their mixture was limited. The triangle used in the design is shown in Figure 4.

The experiment included 14 types of mortars differing in the amount of added fly ash. Mortar types were divided into two groups: air-entrained (AE) and non-air entrained (nAE). The air-entrained mortars were aerated with an admixture of an alkaline alkyl sulfate surfactant (pH = 12.5). The content of chloride in the admixture did not exceed 0.1%, while the content of alkali was less than 2%. The amount of admixture was variable depending on the type and amount of ash added in order to achieve similar air content for each mortar. In fresh air-entrained mortars, the assumed amount of air was 10%, while the actual amount of air in the mortars ranged from 9 to 11%. The water to binder ratio (w/s) was constant at 0.6. The proportions of ingredients in fresh mortars were the following: binder:sand:water = 1:3:0.6. Table 4 shows the quantitative composition of the tested samples. Natural quartz sand with a grain diameter of 0–2 mm from Kopalnia Piasku, Morawica, Poland was used for the mortars. Water was used to make all cement composites in accordance with the requirements of PN-EN 1008 [21].

### 3.4. Compressive Strength

The compressive strength test was carried out after 28 days on samples immersed in water. The test was performed based on the PN-EN 1015-11:2020-04 standard [22] and utilized the KF 300/ACE automatic testing machine (FORM+TEST Seidner&Co. GmbH, Riedlingen, Germany) with a double piston, on six halves of standard bars with dimensions of 40 × 40 × 160 mm from each series.

### 3.5. Linear Strains Measurement

The sulfate attack resistance test was performed in accordance with the procedure contained in the PN-B-19707: 2003 [23] cement standard special cement. Composition, requirements and compliance criteria Annex C: the purpose of the determination of cement resistance to sulfate attack was to establish the resistance of cement to sulfate ions. The test was performed on 6 bars with dimensions of 40 × 40 × 160 mm, stored in a 5% Na_2_SO_4_ solution. For comparison, six bars were immersed in water throughout the entire test period. After 28 days of maturation, the initial length of the samples was measured on a Graff-Kaufman apparatus (AGK, Warsaw, Poland) and immersed in a 5% sodium sulfate solution (STANLAB, Lublin, Poland). In accordance with the standard, the length of the trabecula, l_t_, was measured every 28 days and changes in the external appearance (cracks, scratches, blooms, chippings, etc.) were observed. The sulfate solution was changed every 4 weeks after each measurement.

For each exposure period, the trabecular expansion value was calculated using the formula:

(1) $${\Delta l}_{t}=\left[\frac{\left(l_{t}-{\;l}_{0}\right)}{160}\right]\cdot 1000,\;\left[‰\right]$$

The indication in formula: l_t_—bar length over time t, [mm]; l_0_—bar length after 28 days of maturation, [mm].

### 3.6. The Resistance of Freezing and Thawing Cycles

Mortar resistance on freezing and thawing cycles was tested using the standard method based on the PN-EN 206 + A1:2016-12 standard [24]. This method allows determination of the frost resistance of concrete on the basis of internal and external damage to the sample. This assessment was made on the basis of visual assessment, strength test and on specimens with attached brass plugs for changing the length of the specimens. The test consists of freezing the samples in the air at −18 ± 2 °C for 4 h and thawing in water at +18 ± 2 °C for about 4 h. Before placement to the frost chamber, the mortar samples were impregnated by water until a constant weight was obtained. Every 10 cycles the samples were weighed and the linear deformations were tested with the Graff-Kaufman apparatus.

### 3.7. X-ray Diffraction

The phase composition analysis was performed using the Empyrean two-wheel X-ray diffractometer (Malvern Panalytical Ltd, Grovewood Road, Malvern, WR14 1XZ, United Kingdom). The test samples were hand crushed in a mortar until the desired fineness was obtained. The research was conducted between 5–70° (2θ). The obtained diffraction patterns were analyzed using the ICDD database (International Center for Diffraction Data).

### 3.8. Scanning Electron Microscopy (SEM-EDS)

The analysis of the mortar microstructure was performed using the Quanta FEG 250 FEI COMPANY scanning electron microscope (FEI Company, Eindhoven, The Netherlands). Using the emission of secondary electrons (SE), the topography and morphology characteristics as well as the crystal structure in the tested samples were determined. This analysis was supplemented with a point examination of the elemental composition with the use of the EDAX X-ray analyzer Energy-Dispersive X-ray Analysis (FEI COMPANY, Eindhoven, The Netherlands). Measurements were made under low vacuum (30 Pa) on non-dusted cubic samples about 7 × 7 × 7 mm. Magnifications from 100 to 24,000× were used.

### 3.9. The Air Content in Fresh Mortar

The air content in fresh mortar was determined on the basis of measurements made with the pressure method in accordance with PN-EN 1015 [22]. The vessel was filled up with fresh mortar in four equal layers and compacted by 10 short strokes from the rammer; water was introduced into the chamber to remove all air above the mortar surface. Air was then forced into the sealed chamber and, after the pressure had equalized, the air content was read from a calibrated pressure gauge. The air content was presented as the average value of three measurement tests.

## 4. Results

**Figure 5:**
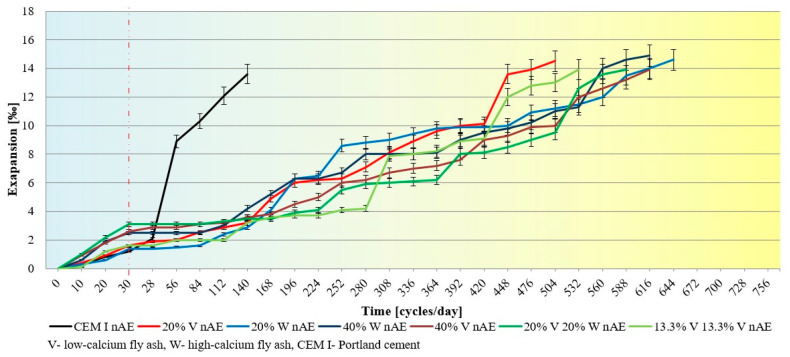
Linear strains measurement of non-air entrained cement mortars subjected to freezing and thawing cycles and sulfate attack.

**Figure 6:**
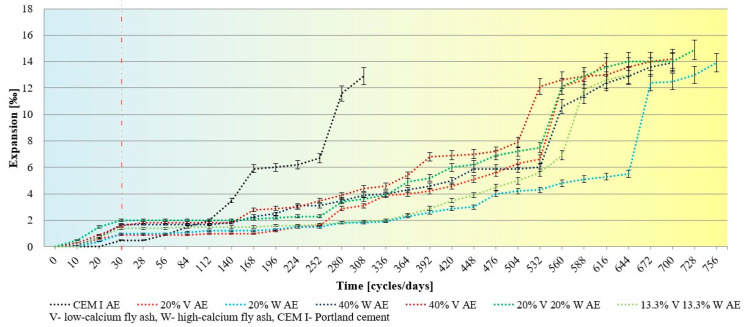
Linear strains measurement of air entrained cement mortars subjected to freezing and thawing cycles and sulfate attack.

The tests of changes in linear strains (expansion) measurement of samples and changes were carried out in the microstructure of the cement slurry in samples that after 30 freezing and thawing cycles were moved to 5% sodium sulfate solution. The compressive strength test was also performed at the moment of moving the samples from the frost chamber, i.e., after 30 cycles, and at the end of the test when the samples showed visual signs of damage caused by sulfate attack. The tests were carried out on 40 × 40 × 160 mm samples. Figure 5 and Figure 6 show linear strains measurement of air-entrained (AE) and non-air entrained (nAE) cement mortars.

The results of the tests of linear deformations of samples after 30 freezing and thawing cycles show minor changes in mortars with the addition of fly ash, although air-entrained Portland cement mortars showed practically no deformations after 30 freezing and thawing cycles. However, after about 140 days of immersion in the sulfate solution of the Portland cement samples, a rapid increase in linear deformations was observed, while in the samples with the addition of fly ash no significant changes in the deformation values were observed. CEMI mortars showed better sulfate resistance after exposure to frost. Air-entrained mortars, in which 20% of Portland cement was replaced with high-calcium fly ash, exhibited the highest durability—the destruction occurred after 760 days in a 5% Na_2_SO_4_ solution. Mortars for which 40% of Portland cement was replaced with both fly ash and their mixture showed similar resistance to the action of the sulfate attack.

**Figure 7:**
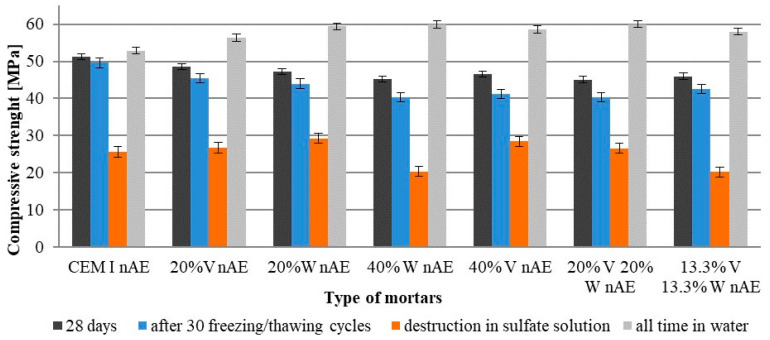
Compressive strength non-air-entrained cement mortars subjected to 30 freezing and thawing cycles and sulfate attack.

**Figure 8:**
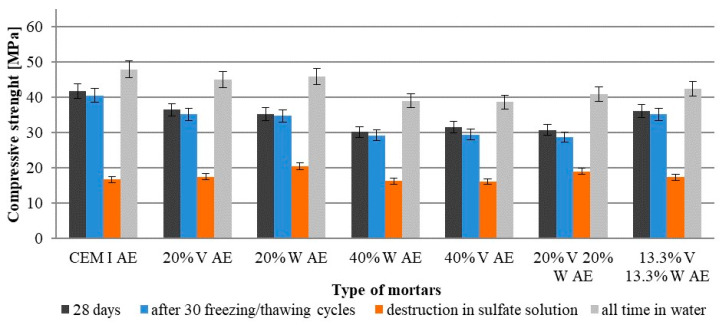
Compressive strength of air-entrained cement mortars subjected to 30 freezing and thawing cycles and sulfate attack.

The compressive strength test of the samples was performed after 28 days of immersion in water, after 30 freezing and thawing cycles, and when the condition of the samples indicated damage caused by sulfate attack. For comparison purposes strength of mortars that were in the water throughout the tests was also analyzed. Figure 7 and Figure 8 show the compressive strength.

After 30 freeze–thaw cycles, the compressive strength of air-entrained Portland cement mortars was practically the same as the strength after 28 days immersed in water. Mortars with 40% Portland cement was replaced by fly ash or their mixture had the greatest decrease in compressive strength as it was translated from freezing–thawing cycles to sulfate sodium solution (amounting to about 10%). The compressive strength after the sulfate attack on these mortars decreased about 50% in relation to the 28-day strength. The compressive strength of mortars which were in water throughout the test period was increased. Mortar with the addition of fly ash had the greatest increase in compressive strength, which proves the course of the pozzolanic reaction.

**Figure 9:**
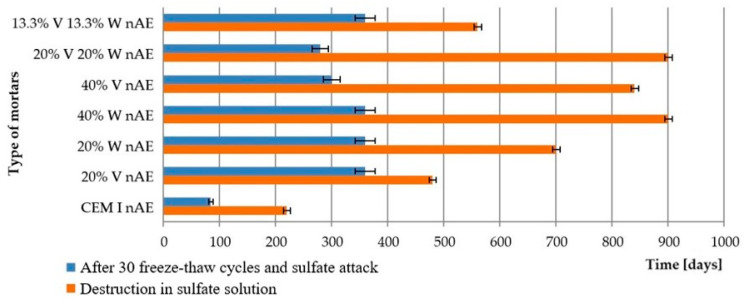
Destruction time of non-air entrained cement mortars after sulfate attack and combined conditions of damage (after 30 freezing/thawing cycles and sulfate attack).

**Figure 10:**
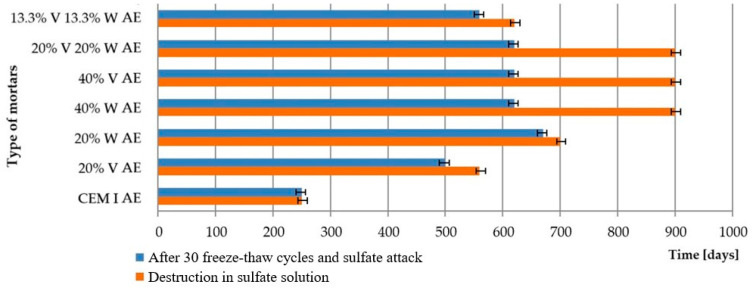
Destruction time of air-entrained cement mortars after sulfate attack and combined conditions of damage (after 30 freezing/thawing cycles and sulfate attack).

In order to determine the durability of the mortars in the combined failure, a comparison was made of the duration required for the destruction of the samples in the sulfate solution alone and in the combined frost and sulfate corrosion damage. The comparison was presented in Figure 9 and Figure 10.

Comparing the linear strain measure, freezing/thawing cycles were accelerating the deterioration of the mortars that were subjected to complex damaging conditions.

The lowest resistance in sodium sulfate solution after prior freezing and thawing was demonstrated by non-air-entrained mortars made of Portland cement. All mortars with fly ash, and also those that had high stability in sulfate solution, showed accelerated damage in 5% Na_2_SO_4_ after cyclic freezing and thawing. Damage and cracks caused by freezing/thawing cycles caused easier access of the solution into the microstructure and faster damage of mortars, which resulted in faster disintegration of the tested samples. The air-entrained Portland cement mortars showed virtually no signs of failure after 30 freeze/thaw cycles and deteriorated after the same residence time in the 5% Na_2_SO_4_ solution as the samples were immersed in 5% Na_2_SO_4_ throughout the test period. Air-entrained mortars with fly ash, subjected to freezing and thawing cycles, followed by sulfate aggregation, as well as Portland cement samples showed better durability than non-air-entrained mortars.

**Figure 11:**
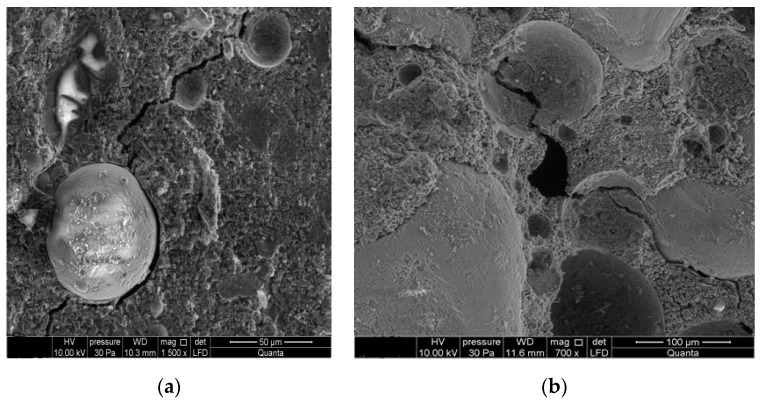
Microstructure of mortars subjected to freezing and thawing cycles: (**a**) with 40% high-calcium fly ash; (**b**) with 40% low-calcium fly ash.

Figure 11 shows a scanning microscope image of cement mortars with fly ash at the moment of transfer from the frost chamber to the sulfate solution. Observing the results of the microstructure indicates clearly visible scratches and cracks formed under freezing-thawing cycles.

On the basis of the microstructure studies carried out by SEM, it was found that the products causing damage were ettringite and gypsum, as in the case of the effect of sodium sulfate attack.

**Figure 12:**
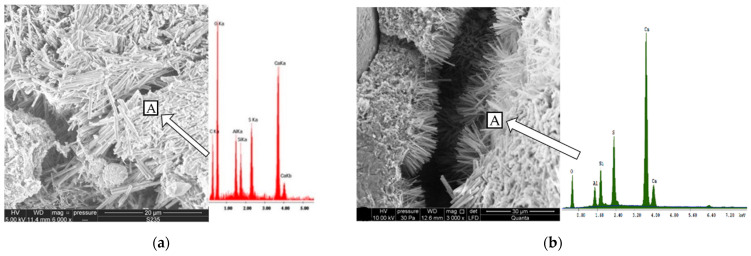
Microstructure of non-air-entrained (**a**) Portland cement mortars (**b**) samples made with 20% low calcium fly ash subjected to 30 freezing/thawing cycles and sulfate attack. A—point when the chemical analyzes was made.

Figure 12 shows the image of damaged samples subjected to 30 freezing and thawing cycles and sulfate attack. Analyzing the photograph of the non-air-entrained Portland cement mortar (CEM I) at the moment of its destruction (Figure 12), a large content of ettringite needles embedded in the C-S-H phase and covering the cracks was visible, most likely formed during the initial freezing/thawing. The morphological diagnosis of ettringite needles is confirmed by the analysis (Figure 12) performed in point A in Figure 12, which showed a high concentration of aluminum and sulfur as well as calcium and silica—components of ettringite and the C-S-H phase.

**Figure 13:**
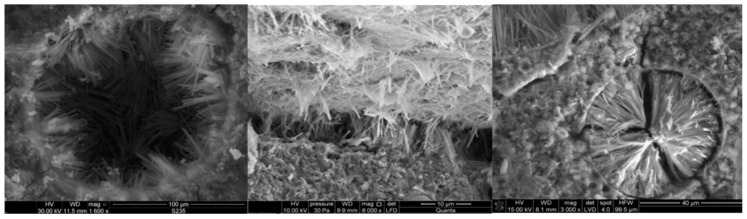
Microstructure of air-entrained mortars subjected to 30 freezing/thawing cycles and sulfate attack. **Left** picture shows fine, loosely arranged ettringite crystals partially filling air pores in air-entrained fly ash mortars. **Center** picture shows the filling the cracks and growing on the inner side of the ettringite cristals. **Right** picture shows the air pore completely filled with ettringite crystals.

In the air-entrained Portland cement mortar, single small cracks and a very large number of densely arranged ettringite crystals partially or completely filling the air pores were observed (Figure 13). In addition to the air pores, clusters of ettringite crystals were also observed. However, no gypsum crystals were observed. The crystals were arranged loosely, not sticking to each other, and they were very small. Minor scratches were observed in all air-entrained mortars initially freezing/thawing. Figure 13 shows fine, loosely arranged ettringite crystals partially filling the cracks and growing on the inner side of the envelopes of air pores in air-entrained fly ash mortars. The highest amount of ettringite crystals was observed in air pores and in cracks, probably formed during freezing/thawing for samples subjected to sulfate attack after the initial frost test.

## 5. Discussion

This study attempted to assess the effect of fly ash on the durability of mortars in more complex destruction conditions, such as freezing and thawing cycles combined with sulfate corrosion. The authors of [21], replacing 20% of cement with fly ash, obtained a better resistance to the action of combined destruction than compared to mortars made of Portland cement. As suggested in [22], replacing 10% OPC with fly ash improved the resistance to combined frost damage and sulfate corrosion. The authors of [24] indicate that fly ash used as the concrete admixture improved the concrete’s resistance against the combined freezing/thawing and sulfate attack with 25% FA a by weight replacement level of cementitious materials, leading to significant improvement in concrete durability, which is also confirmed by our own results. Air-entrained mortars with 20% low-calcium fly ash and 26.6% ash mixture had the best combined failure durability. According to Jiang [25], freezing–thawing cycles and sulfate attacks affected each other and the deterioration of concrete with 20% by weight fly ash replacing OPC attacked by sulfate led to the most aggressive deterioration subject to freezing/thawing cycles—lower sulfate resistance to fly ash upon frost. 

It is well known that the addition of fly ash negatively affects the frost resistance of cement composites; however, an air-entraining additive can be used to enhance the resistance [13,26,27,28,29,30]. The present test results indicate that air-entrained mortars with the addition of fly ash had better resistance under complex deterioration conditions than non-air-entrained mortars made of Portland cement, which is also confirmed by [31,32,33,34]. The introduction of air pores decreased the compressive strength of mortars, as suggested by [35]. One percentage point increase of air content increment may lead to a 2–6% loss of compressive strength [36,37]. The analysis of the compressive strength test results showed a greater decrease in the strength of mortars with the addition of fly ash after adding an air-entraining admixture than in the case of Portland cement. This effect, as suggested by Whiting [36], may be due to the porous structure of the fly ash. A large amount of porous material changes the structure of the cement matrix by introducing additional voids that arise during the application and joining of air bubbles. The authors of [37] showed that the addition of fly ash to air-entrained mortars caused the smaller air voids by eliminating them, which led to a higher number of larger-sized air voids. The authors noticed that in order to aerate the samples with fly ash, the amounts of the air-entraining admixture had to be increased. The authors of [28,38,39] confirmed that achieving the proper level of air entrainment in cement composites containing fly ash requires increasing the amount of air entraining admixture in the concrete. The test conducted by the team of researchers in [40,41] shows that air contents of concrete containing Class C fly ash appear to be more stable than those of concrete-containing Class F fly ash. The higher the organic matter content of fly ash, the higher the air-entraining admixture requirement for concrete in which the admixture is used.

Analyzing the microstructure of cement mortars, it was shown that gypsum and ettringite were the main products of sulfate corrosion, which is also confirmed by the authors’ research in [19], showing that in the case of combined destruction, gypsum was the dominant product. SEM tests showed micro-cracks—the direction and distribution of microcosmic cracks are stochastic, which were formed as a result of freezing and thawing. With the increasing number of cycles, the number of (micro-) cracks has also been observed to increase [42]. A similar relationship was also observed by [17]. Freeze–thaw damage was the main factor which made the bubble inside the concrete become larger and provided a better reaction space for the concrete hydration products and the sodium sulfate reaction. The analysis of the test results showed that air-entrained Portland cement mortars had better sulfate resistance both in sulfate corrosion alone and in the combined failure mode than non-aerated mortars. The positive effect of air entrainment on the sulfate resistance of Portland cement mortars may be related to the presence of large air pores in which the formation of ettringite crystals was observed. The introduction of additional free space in the slurry may favor the free growth of the crystals without creating pressure of crystallization and expansion. The authors of [10] paid special attention to the altered microstructure of the grout around the air pores, which may cause changes in mass transport. The microstructure of the shell air pore (Figure 12a) is characterized by a much more compact structure compared to the adjacent grout. According to Ley [43], the shells are composed of the C-S-H phase with reduced calcium content and no pores are present in them. Despite the high tightness of the shells indicated by the authors [10,43], a relatively large amount of ettringite was observed in the air pores. It seems that this is due to their own permeability and the presence of a permeable transition zone, which was underlined in the publications of Wong [43], but also due to connections with capillary pores which make air pores permeable.

## 6. Conclusions

Considering the natural operating conditions in which cement composites are exposed to the effect of sulfate solutions, and the cyclic freezing and thawing, it is recommended to use up to 20% appropriate air-entrained high-calcium fly ash as binder additive.

Better resistance to the combined frost damage and sulfate corrosion was exhibited by aerated mortars with the addition of 20% calcium ash. Addition of air pores significantly improved the resistance to combined damage.

The durability of non-air-entrained mortars with 40% addition of fly ash or their mixtures, which exhibited good resistance to sulfate corrosion, decreased markedly when they were exposed to combined frost damage and sulfate aggression. The preliminary frost damage reduced the resistance to the effect of sulfate solution.

A product of the sulfate attack, such as ettringite, was observed in the air pores, which explains the better resistance to the action of sodium sulfate in air-entrained mortars. The ettringite crystals had large shaping surfaces, therefore the strains of air-entrained samples were much smaller in size than those of the non-air entrained mortars.

## Figures and Tables

**Table 1:** Chemical composition of Portland clinker of the tested cements [%].

Cement	SiO_2_	Al_2_O_3_	Fe_2_O_3_	CaO	MgO	SO_3_	Cl^−^	Na_2_O_eq_
CEM I	20.6	5.75	3.4	65.8	1.5	2.5	0.014	0.77

**Table 2:** Phase composition of Portland clinker of the tested cements [%].

Cement	C_3_S	C_2_S	C_3_A	C_4_AF
CEM I	63.2	8.2	6.3	9.9

**Table 3:** Chemical composition of low and high-calcium fly ash used in mortars [%].

Fly Ash	SiO_2_	Al_2_O_3_	Fe_2_O_3_	CaO	MgO	SO_3_	Cl^−^	Na_2_O	K_2_O
Low- calcium	50.5	26.1	7.4	4.5	2.9	0.5	0.01	-	-
High- calcium	45.2	20.8	4.6	20.6	1.5	3.0	0.014	0.23	0.19

**Table 4:** Weight composition of the tested mortars.

Mortars Composition [%]	Cement, C [g]	Low-Calcium Fly Ash, V [g]	High-Calcium Fly Ash, W [g]
CEM I = 100% C	500	0	0
20% V = 80% C + 20% V	400	100	0
40% V = 60% C + 40% V	300	200	0
20% W = 80% C + 20% W	400	0	100
40% W = 60% C + 40% W	300	0	200
20% V 20% W = 60% C + 20% V + 20% W	300	100	100
13.3% V 13.3% W = 73.3% C 13.3% V 13.3% W	370	65	65

## Data Availability

Not applicable.
